# The insulin centennial—100 years of milestones in biochemistry

**DOI:** 10.1016/j.jbc.2021.101278

**Published:** 2021-10-28

**Authors:** Alan D. Attie, Qi-Qun Tang, Karin E. Bornfeldt

A hundred years ago, Frederick Banting and Charles Best, in the laboratory of John MacLeod at the University of Toronto, performed a historic experiment demonstrating that an extract from pancreata in which the pancreatic ducts had been ligated to allow isolation of the “internal secretion” lowered blood glucose in dogs. This experiment broke ground for a fertile field that now extends into essentially all branches of physiology, endocrinology, biochemistry, cell biology, genetics, and molecular biology. This centennial is being celebrated by the retelling of this extraordinary story and the research triumphs that mark the trajectory to the present. Several journals have already paid tribute to these events (1, 2, 3, 4, 5).

We wish to highlight the role of our two ASBMB journals, *The Journal of Biological Chemistry* and *The Journal of Lipid Research* in publishing impactful biochemical studies in this field. We have selected 27 articles for republication. The choice of so few articles from a vast literature was very difficult and somewhat arbitrary, but it allows us to highlight a subset of the many great discoveries that advanced our understanding of insulin and to recognize the remarkable insights of early pioneers of this field. Many of the most important articles in the field were published in other journals and are therefore not included in this series.

What comes across in reading the earlier publications in this field is how prescient were the insights and speculations of the brilliant scientists who launched and advanced this field. In threading together these stories, we have gained a new appreciation of the hard work, zealous dedication, and brilliant insights of the scientists involved in this quest. Although diabetes is principally defined by hyperglycemia, the role of lipids in diabetes has been a dominant theme in this field and we point to several highlights in this story.

## Insulin purification

With the realization that a substance produced in the pancreas is capable of lowering blood glucose and is likely missing in people with diabetes, it became critical to purify and identify this substance. The first purification of insulin, from the cow pancreas, was performed by the Canadian biochemist James Collip and was used to treat the first human patient with diabetes, Leonard Thompson, in 1922.

American scientists were also working on purifying insulin. The first article on insulin that was published in the JBC is a study of precipitation methods by Kimball and Murlin in 1923 (6). They tested a series of alcohols, acetone, ether, toluene, xylene, trichloroacetic acid, and several salts. The method was to centrifuge the precipitate, resuspend in water, and immediately inject into rabbits. What is most remarkable about the article is how inconclusive it was regarding insulin’s properties. It did not promote any one precipitation method and ended by saying, “With regard to the properties of insulin as it has been observed in this laboratory, not much can be said. It is a white, amorphous powder probably insoluble in neutral water when pure. It gives no protein reactions of any kind, and the most potent that have been analyzed have had a low nitrogen content, 4 to 6% dry weight.” The real significance of that article (6) by Kimball and Murlin, however, was the discovery of glucagon, which they described as a “hyperglycemic substance” in the pancreatic extracts used to purify insulin.

Patients with diabetes went on to be treated with insulin purified from cattle or pigs. However, with the development of a radioimmunoassay by Rosalyn Yalow and Solomon Berson (7), it became clear that many patients developed antibodies against bovine and porcine insulin, making it less effective with time. Human insulin was not to become available until late in the 1970s, when the first genetically engineered insulin was produced in *Escherichia coli* by scientists at Genentech (8). Rosalyn Yalow became the second woman to receive the Nobel Prize in Physiology or Medicine for her work. The first was Gerty Cori (see below).

Berson and Yalow quickly realized that patients with type 2 diabetes were most often not insulin-deficient and in fact many had hyperinsulinemia. They anticipated the intense focus on insulin resistance in this field with this comment in their famous publication describing the insulin radioimmunoassay (9): “appreciation of the lack of responsiveness of blood sugar, in the face of apparently adequate amounts of insulin secreted by early maturity-onset diabetic subjects, is obviously of importance in the interpretation of the pathogenesis of this type of diabetes. However, the data at hand can only indicate that absolute insulin deficiency *per se* is not the cause of the hyperglycemia and suggest other possibilities that merit investigation, namely (1) abnormal tissues with a high threshold for the action of insulin; (2) an abnormal insulin that acts poorly with respect to hormonal activity *in vivo* but reacts well immunologically *in vitro*; (3) an abnormally rapid inactivation of hormonally active sites … but not of immunologically active sites on the insulin molecules; and (4) the presence of insulin antagonists. The last suggestion has been made many times by previous workers. A joint attack on the problem, utilizing both the specific immunoassay for plasma insulin and an assay method that measures the net biological effect of insulin and its inhibitors would seem to be indicated.”

## Processing of proinsulin

A pioneer in the study of post-translational processing of insulin was Don Steiner. He narrates this story in a lovingly written JBC article, published in 2011 (10). The determination of the sequence and structure of insulin by Fred Sanger in 1955 (11) (who received two Nobel Prizes for his work on protein and DNA sequencing) begged the question, are the two chains of the insulin molecule derived from a common precursor? In 1967, Don Steiner published his seminal discovery, through protein purification and pulse-chase experiments in isolated islets, of a precursor to insulin, proinsulin (12). Proinsulin consists of the insulin A and B chains connected by a peptide (C-peptide), which is cleaved off in the secretory granules before secretion of mature insulin. Steiner also recognized the utility of the C-peptide as a quantifiable marker of insulin production and β-cell function, and developed its assay in 1970 (13). Steiner went on to discover several of the enzymes that process proinsulin to mature insulin. This work has had a profound impact on cell biology and endocrinology because many protein hormones, clotting factors, growth factors, receptors, and even serum albumin are synthesized as a precursor with a propeptide that is removed during its transport through the secretory pathway.

The 3D structure of mature porcine insulin was solved by Dorothy Hodgkin (14), who had applied X-ray crystallography to reveal 3D structures of several other molecules before that. She was awarded the Nobel Prize in Chemistry in 1964 for her discoveries. Not surprisingly, the 3D structure of insulin is critical for its biological activities.

Graeme Bell reported in 2007 that mutations in the insulin gene that cause abnormalities in insulin maturation and folding cause diabetes (15). Protein misfolding activates a transcriptional program, the unfolded protein response, which can ultimately lead to cell death and is now recognized as a major cause of diseases caused by missense mutations in proteins, ranging from neurological diseases to metabolic diseases.

Jeremy Thorner’s discovery of the yeast protease that processes the yeast mating factor, Kex2p, led to the discovery of the convertases that process proinsulin and proglucagon (16) and then to an entire family of proprotein convertases (17). Many endocrine disorders and some obesity syndromes are caused by mutations in these enzymes. Thus, the discovery of processing of proinsulin to insulin spurred a number of seminal subsequent discoveries.

## Insulin secretion

The pathways that link glucose sensing with insulin secretion have been at the heart of islet biology for decades. A milestone in the field was the 1984 discovery by Frances and Stephen Ashcroft at the University of Oxford that ATP-sensitive potassium (KATP) channels link ATP generation to insulin secretion (18). Metabolism of glucose in the β-cell leads to a rise in ATP, the closure of the KATP channel, and the activation of voltage-gated Ca^2+^ channels, leading to Ca^2+^ influx and insulin secretion. The KATP channel is an octamer consisting of two proteins, Kir6.1 (KCNJ11) or Kir6.2 and the sulfonylurea receptor, SUR1 (ABCC8) or SUR2. Loss-of-function mutations in SUR1 or less commonly, KCNJ11, lead to hyperinsulinism (19), whereas mutations in KCNJ11 that decrease ATP inhibition of KATP cause neonatal permanent neonatal diabetes (20).

Glucokinase plays a role in glucose sensing in the liver and β-cells. In the liver, glucokinase expression is regulated by insulin, and its abundance determines the capacity of the liver to metabolize glucose, which, unlike the muscle and adipose tissue, is rate-limiting for its uptake. Matschinsky and Ellerman discovered in 1968 that glucokinase is present in β-cells (21). The discovery of genes causing monogenic dominantly inherited diabetes syndromes, termed maturity-onset diabetes of the young, provided valuable mechanistic information about β-cell biology (22). Specifically, loss-of-function mutations in glucokinase lead to diabetes (23), whereas the rarer gain-of-function mutants cause hyperinsulinism (24). This supports the role of glucokinase as a β-cell glucose sensor that determines the capacity of the β-cell to take up glucose and oxidize it in the glycolytic pathway and led to the development of glucokinase activators as a potential treatment for diabetes (25). Other maturity-onset diabetes of the young genes include transcription factors that play roles in β-cell development and in the function of the adult β-cell.

Although glucose is the best-studied insulin secretagogue, amino acids can also stimulate insulin secretion. Gain-of-function mutations in glutamate dehydrogenase cause excessive amino acid–induced insulin secretion (26). The product of glutamate dehydrogenase is mitochondrial α-ketoglutarate, raising the question how this metabolite could signal insulin secretion. An important clue came from the discovery that β-cells have an abundance of mitochondrial phosphoenolpyruvate carboxykinase, encoded by the *PCK2* gene. Deletion of the gene in mice leads to a severe defect in insulin secretion (27) as shown by Richard Kibbey’s laboratory at Yale University. Subsequent studies have proposed that formation of phosphoenolpyruvate promotes cycling through the pyruvate kinase reaction and this is intimately connected with the closure of the ATP channel (28, 29).

Unlike most other cell types, pyruvate metabolism in β-cell mitochondria partitions fairly equally between the anaplerotic route (pyruvate carboxylase) and the oxidative route (pyruvate dehydrogenase), as demonstrated by Schuit and colleagues (30). Much of the product, citrate and/or isocitrate, exit the mitochondria. As in other cell types, malonyl-CoA gates fatty acid entry into the mitochondria and subsequent β-oxidation by inhibiting carnitine palmitoyl transferase-1. Cytosolic citrate gives rise to acetyl-CoA and then malonyl-CoA. Cytosolic isocitrate can be oxidized to α-ketoglutarate, giving rise to reducing equivalents in the form of NADPH. NADPH has been suggested to play a role in amplifying insulin secretion. One pathway, proposed by Newgard and MacDonald, suggests that isocitrate dehydrogenase-2 in the cytosol produces NADPH, which reduces glutathione, and this activates the de-SUMOylating enzyme sentrin/SUMO-specific protease-1, which has as its substrate tomosyn-1, which regulates syntaxin 1A during insulin exocytosis (31, 32).

Fatty acids amplify insulin secretion. Blocking fatty acid oxidation enhances insulin secretion. Prentki has proposed a model whereby monoglycerides, primarily derived from hydrolysis of triglycerides, bind to Muc18 and stimulate insulin exocytosis (33). Fatty acids also bind to a G-protein–coupled receptor, FFA1/Gpr40, which may amplify insulin secretion through an independent route (34).

## Incretin hormones

Beginning as early as 1902, it was postulated that the intestine produces factors that lower blood glucose. This was refined into the term “incretin” by La Barre in 1932 and was followed by numerous studies with intestinal extracts, which supported and then failed to support the role of an intestinal extract in glucose regulation (reviewed in (35)). The development of the insulin radioimmunoassay by Yalow and Berson facilitated the search for incretins by directly measuring insulin secretion. The incretin concept was proposed in 1964 based on the observation of a much greater insulin response to an oral glucose than an intravenous glucose challenge (36, 37). The first incretin hormone to be purified was glucose-dependent insulinotropic polypeptide, and its action in humans was first demonstrated in 1973 (38).

The mammalian preproglucagon cDNA was cloned in 1983 by Graeme Bell. He showed that the gene encoded glucagon, the incretin glucagon-like peptide-1 (GLP1) and glucagon-like peptide-2 (39). The GLP1 receptor was cloned in 1992 by Bernard Thorens (40). The ground-breaking work of Habener, Holst, Drucker, and many others laid the foundation for GLP1 agonists as antidiabetic drugs. The discovery of exendin-4, isolated from *Heloderma suspectum* venom, provided a highly effective stable GLP1 agonist (41). The development of inhibitors of a circulating protease that degrades GLP1, dipeptidyl peptidase-4 by Nancy Thornberry and Ann Weber, delivered an orally available alternative to injected GLP1 agonists (42). Recent work has also shown that glucagon can function in a paracrine fashion to stimulate insulin secretion through the GLP1 receptor (43).

GLP1 has numerous actions unrelated to glycemic control, including effects on the heart rate, inflammation, blood pressure, hepatic steatosis, and food intake. In addition to its production by intestinal L-cells and pancreatic α-cells, GLP1 is synthesized in the central nervous system where it regulates food intake (reviewed in (44)).

## The discoveries that insulin acts by increasing glucose uptake into cells

Levine and Goldstein published a landmark article in the JBC in 1949 (45), with just one page of text and one figure [see Fig. 1 in (45)]. This report is generally regarded as the first evidence that insulin stimulates hexose uptake into cells. Before 1949, many scientists believed that insulin acted by entering cells and interacting with cellular enzymes to change their activity and downstream glucose metabolism. Levine and Goldstein used galactose as a nonmetabolized sugar to follow its fate 30 min after injection of insulin into a dog. After observing an increase in the disappearance of galactose from the circulation, they presciently concluded, “The working hypothesis prompted by these data can be stated as follows: Insulin acts upon the cell membranes of certain tissues (skeletal muscle, etc.) in such a manner that the transfer of hexoses (and perhaps therapeutic substances) from the extracellular fluid into the cell is facilitated. The intracellular fate of the hexoses depends upon the availability of metabolic systems for their transformation. In the case of galactose, no further changes occur. In the case of glucose, dissimilation, glycogen storage, and transformation to fat are secondarily stimulated by the rapidity of its entry into the cell.”

In 1961, Charles “Rollo” Park reported in the JBC that the rate-limiting step in the stimulation by insulin of glucose utilization by the perfused heart is transport across the plasma membrane (46). Remarkably, he also discovered that anoxia stimulated glucose transport, anticipating the later work on exercise and AMPK-stimulated glucose uptake into muscle.

The mechanism by which insulin stimulates glucose uptake into cells was revealed in the late 1980’s when the main insulin-sensitive glucose transporter, GLUT4 (gene name *SLC2A4*), was cloned by a group led by Graeme Bell and Susumu Seino at the University of Chicago (47) and by David James and Mike Mueckler (48). It was later shown by Sam Cushman and Tetsuro Kono that insulin promotes the translocation of GLUT4 to the plasma membrane (49, 50). Subsequent work of Jeffrey Pessin’s group (51) and others (52) showed that insulin-stimulated translocation of GLUT4 vesicles from intracellular stores to the plasma membrane engages the exocytotic machinery of the cell, that is, some of the same cellular machinery required for insulin exocytosis in the β-cell.

The translocation of GLUT4 to the plasma membrane is not exclusively stimulated by insulin, however. In a highly impactful study, Amira Klip reported in 1990 in the JBC that exercise can also induce GLUT4 translocation to the plasma membrane (53).

## Role of insulin in glycogen metabolism

As early as the 1920s, Carl and Gerty Cori, working in Buffalo, NY, established the ‘cycle of carbohydrate’ aka the Cori Cycle. Blood glucose is converted into muscle glycogen, as an energy storage mechanism. Glycogen can then be metabolized back to glucose (through glycogenolysis) and be used to generate ATP through glycolysis and mitochondrial oxidation. However, during strenuous exercise, due to the lack of sufficient oxygen, glycolysis generates lactic acid, which is released into the blood. Lactic acid is converted back to glucose in the liver through gluconeogenesis and can then be used by muscle. The Coris discovered that insulin accelerates the cycle from the liver to muscle (54). Carl and Gerty Cori were awarded the Nobel Prize in Physiology or Medicine in 1947 for their discoveries.

The liver is the second important glycogen storage tissue. In the liver, the first step in glucose conversion to glycogen, phosphorylation of glucose to glucose-6-phoshate, is catalyzed by glucokinase. Chernick and Chaikoff, long before the discovery of the activation of glucokinase by insulin (55), proposed that there was a block in glucose transport between glucose and glucose-6-phosphate in liver slices from diabetic rats (56). James Ashmore’s laboratory at Harvard showed in 1955 that insulin had only modest and delayed effects on carbohydrate metabolism in liver slices as compared with its rapid (within minutes) effects in muscles (57). Thus, it was already known in the 1950s that the rate-limiting step for glucose utilization differed in the muscle and liver.

## Insulin stimulates lipogenesis

The liver is the only organ that synthesizes fatty acids and triglycerides for export to other tissues, such as adipose tissue, where triglycerides are stored in lipid droplets until needed. In 1944, Stetten and Boxer used tracer studies with deuterated water to discover that diabetic animals were deficient in their ability to convert glucose into lipids (58). Six years later, Chernick *et al.* (59) demonstrated that this deficiency was present in liver slices from diabetic rats, showing for the first time that the tissue retained its “memory” of the insulin deficiency of the intact animal. Many years later, the Brown and Goldstein laboratory discovered that insulin increases the expression of SREBP-1c, the master transcriptional regulator of genes encoding lipogenic enzymes (60) in a pathway that requires the activity of the mechanistic target of rapamycin complex 1 (61). Insulin also stimulates lipogenesis in adipose tissue, which together with insulin-mediated suppression of lipolysis contributes to the weight gain in response to insulin treatment.

## Mechanism for insulin suppression of ketogenesis

The earliest characterizations of patients with type 1 diabetes and animal models with surgical or chemical ablation of insulin-producing cells observed life-threatening ketosis. Ketosis, the excess generation by the liver of ketone bodies when the body does not have a sufficient supply of carbohydrates, can lead to ketoacidosis. The mechanism for insulin’s inhibition of ketogenesis was first characterized by the studies of J. Denis McGarry. First, working with Dan Foster in 1971, McGarry noted that ketogenesis from the straight-chain fatty acid octanoate, which is not used as a substrate for triglyceride synthesis, occurred at a much faster rate in fasted rats than in fed rats (62). Moreover, ketogenesis from octanoate occurred at a maximal rate in diabetic rats. The group concluded that an accelerated generation of acetyl-CoA was responsible for the increased ketogenesis in diabetes. McGarry and Foster went on to discover that there was increased carnitine in diabetic livers and increased flux of fatty acids through the carnitine acyl-transferase reaction (63), leading to the increased acetyl-CoA levels and subsequent ketogenesis.

The site of inhibition of ketogenesis by insulin was unknown at this time. Richard Veech *et al.* had observed that malonyl-CoA levels are decreased in response to glucagon, diabetes, and starvation and increased in response to insulin. Glucose had been known to suppress ketogenesis. This set McGarry and Foster on an 18-month quest for an intermediate in glucose metabolism that could inhibit ketogenesis in liver homogenates. Inspired by the simultaneous regulation of glycogen synthesis and breakdown, they expanded their search to look for a molecule that could regulate fatty acid synthesis and oxidation and landed on malonyl-CoA (64). McGarry and Foster discovered that malonyl-CoA is a potent regulator of carnitine acyl-transferase I and thus gates the entry of substrate fatty acid into the mitochondria, controlling fatty acid β-oxidation and ketogenesis (64). They went on to show that glucagon simultaneously blocks fatty acid synthesis and stimulates ketogenesis while regulating the level of malonyl-CoA (65). With the prior knowledge that insulin and glucagon regulate acetyl-CoA carboxylase, McGarry and Foster were able to deduce that this enzyme is the key locus for the effect of these two hormones on ketogenesis, through the production of malonyl-CoA and its inhibitory effect on mitochondrial carnitine palmitoyltransferase I. Through this mechanism, insulin suppresses ketogenesis by limiting access of fatty acids into mitochondria for generation of acetyl-CoA and subsequent ketogenesis.

## Mechanism for insulin suppression of lipolysis in adipocytes

Before the discovery of insulin, children with type 1 diabetes became emaciated and often died within a year of diagnosis. This dire disease progression changed rapidly with insulin treatment of the first patients. In a letter to Banting from one of the first patients treated with insulin, Teddy Ryder wrote “I wish you could come to see me. I am a fat boy now and I feel fine.”

The mechanism whereby insulin promotes adiposity by suppressing lipolysis remained elusive for decades. The mechanism was finally elucidated in large part by Earl Sutherland, Jr, who was awarded the Nobel Prize in Physiology or Medicine in 1971 for his discovery of the second messenger cyclic AMP. Sutherland had trained with the Coris in the early 1940s. In a milestone article published in the JBC in 1966, he concluded that “the antilipolytic action of insulin is due at least in part to the suppression of cyclic AMP levels” and that “it appears most likely that insulin is inhibiting the synthesis of cyclic AMP or possibly accelerating its inactivation” (66). We now know that insulin acts to suppress lipolysis by activating a cyclic nucleotide phosphodiesterase (PDE3B), which converts cyclic AMP to 5′-AMP (67).

By lowering cyclic AMP levels, insulin causes a reduced activity of PKA (also known as cAMP-dependent protein kinase). Greenberg and Egan, working in the laboratory of Constantine Londos, identified an important phosphoprotein substrate of PKA in 1990 (68, 69). They termed the protein perilipin as it coats lipid droplets. Perilipin sequesters alpha-beta hydrolase domain 5, *aka* CGI-58, prevents the interaction of alpha-beta hydrolase domain 5 with adipose triglyceride lipase, and reduces the first step in lipolysis, the hydrolysis of triglycerides to diacylglycerols (70, 71, 72). PKA also targets hormone-sensitive lipase to the surface of lipid droplets and enhances its lipolytic activity (73, 74). Together, the action of insulin is to strongly suppress lipolysis in adipose tissue.

## Discovery of the insulin receptor and its tyrosine kinase activity

The 1970s witnessed the discovery of the kinase cascades that respond to insulin signaling. The Larner laboratory led the way in discovering the role of cyclic AMP in the regulation of glycogen metabolism (75). Joseph Larner’s group further showed that insulin could promote the activation of glycogen synthase through its inhibition of glycogen synthase kinase (75). These studies provided a template for later work that identified other protein kinases that could modulate these enzymes.

By this time, it was well appreciated that insulin’s actions are mediated by its receptor. Already in the early 1950s, Martha Vaughan had provided seminal discoveries indicating the presence of an insulin receptor. In a JBC article published in 1952, she labeled insulin with ^131^I and ^35^S and analyzed its binding to isolated rat diaphragms to show that “insulin is bound by chemical bonds of as yet undetermined character to tissue” (76). A couple of years later, she showed that labeled insulin also binds to typical insulin target tissues (liver, adipose tissue, skeletal muscle) *in vivo* in rats and dogs (77). Pedro Cuatrecasas later showed that the primary action of insulin is at the cell membrane (78) and then used affinity chromatography and detergent solubilization methods to purify the insulin receptor from liver cell membranes (79). Ronald Kahn, David Neville, and Jesse Roth carried out the first receptor-binding assays and concluded that the levels of the insulin receptor are reduced in obese animals, providing a plausible explanation for insulin resistance (80). This was long before the regulation of downstream signaling events were studied.

In 1980, Pilch and Czech, by cross-linking insulin with its receptor and visualizing it by gel electrophoresis under reducing and nonreducing conditions, concluded that the receptor is made up of proteins linked by disulfide bonds (81). A short time later, Hedo *et al.* (82) went on to show that in fact the chains of the receptor are derived from a single polypeptide precursor that is proteolytically cleaved to give rise to its two chains, which remain connected by a disulfide bond and assemble into an α2β2 heterotetramer.

The discovery by Kasuga *et al.* (83) in Ronald Kahn’s laboratory that the insulin receptor is a protein tyrosine kinase that catalyzes its own phosphorylation inspired detailed mechanistic exploration of its other substrates and the cascade of events that is triggered when insulin binds to its receptor. Through mutagenesis experiments, Ora Rosen demonstrated that the tyrosine kinase activity of the insulin receptor is essential for its effects on glucose uptake and metabolism, and its mitogenic effects, but not for binding to insulin (84). More precisely, the autophosphorylation of the receptor regulates its kinase activity in a feed-forward fashion, as shown by Morris White while working in the Kahn laboratory (85, 86). The many remaining unanswered questions in this field are a testament to the immense complexity of the insulin receptor signaling pathway and cross-talk with other intracellular signaling pathways.

## Insulin receptor kinase substrates, a gateway into the insulin signaling pathway

In 1987, White *et al.* (87) reported the purification of a soluble protein in cells that is phosphorylated by the insulin receptor; it came to be known as insulin receptor substrate 1 (IRS1). At least eight additional substrates of the insulin receptor and the insulin-like growth factor receptor-1 were subsequently identified. The autophosphorylation of the insulin receptor enables it to recruit its substrates. IRS1 and IRS2 were then shown to recruit other essential components of the insulin signaling cascade. The early 1990s saw the rapid expansion of research into the insulin receptor signaling pathways. Among the most important discoveries were Lewis Cantley’s discovery of PI3K, which catalyzes the formation of phosphatidylinositol-3-phosphate (88) and the subsequent demonstration that insulin receptor activation results in activation of PI3K (89). With the identification of the serine–threonine kinase Akt by Philip Tsichlis (90) and the finding that a constitutively active Akt mutant mimics the action of insulin on glucose uptake by Morris Birnbaum and Richard Roth (91), Philip Cohen’s group discovered the serine–threonine kinase PtdIns(3,4,5)P3-dependent protein kinase-1 as the link between PI3K and Akt activation (92).

Insulin receptor–induced activation of Akt explains many of the metabolic effects of insulin. Accordingly, Akt isoforms have numerous substrates, including members of the FOXO family of transcription factors, as beautifully illuminated by Domenico (Mimmo) Accili (93). FOXO1 activates genes in gluconeogenesis and, when phosphorylated by Akt, is excluded from the nucleus, providing a basis for insulin’s inhibition of gluconeogenesis at the transcriptional level. Akt also activates PDE3B (67), explaining in part the ability of insulin to decrease cyclic AMP levels and inhibit lipolysis discussed above. Akt phosphorylation of its substrate GSK3 (glycogen synthase kinase 3) inactivates this kinase, contributing to the dephosphorylation and activation of glycogen synthase and to an acceleration of glycogen synthesis (94). John Lawrence demonstrated that Akt can act upstream of mechanistic target of rapamycin (95) and that this mechanism contributes to insulin’s ability to stimulate glycogen synthesis (96). However, other signaling events contribute significantly to insulin-mediated glycogen synthesis. Research in Alan Saltiel’s laboratory revealed that activation of a protein phosphatase (PP1) is more critical than GSK3 in mediating glycogen synthesis in adipocytes (97). Moreover, the insulin-induced GLUT4 translocation from intracellular stores to the plasma membrane, necessary for insulin’s ability to stimulate glucose uptake, depends on a number of small GTPases, some of which act downstream of Akt (98, 99).

In addition, research in several laboratories soon revealed that insulin receptor activation induces intracellular signaling largely distinct from the PI3K-Akt pathway. For example, research by the laboratories of Thomas Sturgill, Jeffrey Pessin, and others revealed that some nonmetabolic actions of insulin, such as mitogenesis and cell adhesion, are explained by activation of the ERK pathway (100, 101, 102, 103).

The development of Cre recombinase-LoxP technology to generate mice with cell type–selective gene targeting allowed Ron Kahn, Barbara Kahn, Rohit Kulkarni, and others to study tissue-selective effects of the insulin receptor. The first such mouse model was a skeletal muscle–targeted insulin receptor–deficient mouse, which surprisingly exhibited elevated fat mass and plasma lipid levels without altered blood glucose levels, highlighting the role of insulin in suppression of plasma lipid levels and the redistribution of substrates from skeletal muscle to adipose tissue (104, 105). Many more tissue-targeted insulin receptor–deficient models followed, including a β-cell–targeted insulin receptor–deficient model, which showed an insulin secretory defect (106), a liver-targeted model, which showed severe glucose intolerance (107), and an adipose tissue–selective insulin receptor knockout, which exhibited reduced fat mass and increased longevity (108).

Together, these studies further emphasize the complexity of insulin receptor signaling and the ability of the organism to shift substrate utilization and to compensate for tissue-selective insulin receptor loss. It should be noted, however, that whole-body deletion of the insulin receptor is not compatible with life in the mouse, and these animals die from ketoacidosis 2 to 3 days after birth (109).

## Discoveries related to insulin resistance and type 2 diabetes

Although patients with type 1 diabetes were the first to benefit from the discovery of insulin, in 1936, Sir Harold Himsworth suggested that diabetes can be classified into insulin-sensitive and insulin-insensitive types (110). We now know that type 2 diabetes is by far the more prevalent form of diabetes and that type 2 diabetes is preceded by insulin resistance. The understanding that type 2 diabetes often is preceded by and associated with insulin resistance fueled much research into the causes of insulin resistance, including the question of which comes first, hyperinsulinemia or insulin resistance? (111)

The 1970s saw the identification by Jeffrey Flier of anti-insulin receptor antibodies as the cause of severe insulin resistance in identified patients with an unusual diabetic syndrome (112). Jerrold Olefsky showed that insulin resistance could be explained by both defects in the insulin receptor and postreceptor defects (113), and Takashi Kadowaki in Simeon Taylor’s group identified insulin receptor mutant alleles in a patient with severe insulin resistance (114). Better methods of estimating insulin sensitivity in patients, based on mathematical modeling, were developed by Richard Bergman (115, 116).

With the increased understanding of the insulin receptor signaling pathways came the realization in the early 2000s that phosphorylation of specific insulin receptor substrates and cross-talk with other signaling pathways can contribute to insulin resistance. Shoelson and White, who both had trained with Ron Kahn, published articles in the JBC demonstrating that phosphorylation of serine 307 in IRS1 blocks its interaction with the insulin receptor (117, 118). One of the serine/threonine kinases found to be responsible for phosphorylation of two IRS1 sites, Ser302 and Ser307, was c-Jun N-terminal kinase, a kinase activated by cytokines, such as TNF-α (119). These findings contributed to the development of the currently very active research area of immunometabolism, which investigates the many links among inflammation and metabolism (120).

## Interplay between lipid metabolism and insulin sensitivity

Obesity and hepatic steatosis are often accompanied by insulin resistance. This led some investigators to infer that hepatic steatosis causes insulin resistance. However, a closer look showed in several models of hepatic steatosis that triglyceride accumulation in hepatocytes by itself does not cause insulin resistance (121, 122). These models affect lipogenesis, fatty acid oxidation, or the release of free fatty acids from adipose tissue. For example, in a *JLR* article published in 2008, it was shown that deletion of microsomal triglyceride transfer protein has no effect on hepatic or peripheral insulin sensitivity despite a 7-fold increase in liver triglycerides (122). Likewise, Robert Farese’s group showed that overexpression of diacylglycerol acyltransferase-2 leads to hepatic steatosis, but not insulin resistance (123). These findings support the notion that triglycerides, when stored in lipid droplets in cells, are largely harmless.

Dissociation of triglyceride accumulation from insulin resistance led to investigation of other lipids (diglycerides and ceramides) as possible causal culprits in insulin resistance (122). Shulman *et al.* advanced the idea that diacylglycerols are key players in insulin resistance, through their ability to stimulate a specific isoform of PKC (PKC_ε_), which then phosphorylates T1150 in the catalytic domain of the insulin receptor, inhibiting its tyrosine kinase activity. Recently, Shulman refined his model by proposing in a recent *JLR* article that it is specifically the plasma membrane pool of diacylglycerol that mediates insulin resistance (124).

An alternative mechanism for lipid-mediated suppression of insulin signaling has been advanced by Scott Summers. His model is that palmitate, as a substrate, increases the abundance of ceramide, which then blunts insulin signaling by decreasing the pool of activated Akt (125), by increasing the dephosphorylation of Akt, and by blocking its translocation to the plasma membrane. Summers obtained *in vivo* proof of principle when his team deleted the enzyme dihydroceramide desaturase 1 in mice and found a marked improvement in glucose tolerance, insulin sensitivity, and amelioration of hepatic steatosis in leptin-deficient animals (126).

Barbara Kahn discovered that modulation of GLUT4 expression in adipose tissue results in changes in whole-body insulin sensitivity. This led her team to discover molecules originating in adipose tissue that signal to other tissues (127). Working with Alan Saghatelian, Kahn identified a new class of bioactive lipids consisting of a fatty acid with a second fatty acid chain attached through an ester linkage. Kahn has demonstrated that in addition to their insulin-sensitizing effects, these compounds have anti-inflammatory activity and are able to stimulate glucagon-like peptide release from entero-endocrine cells (128).

Together, these discoveries further highlight the important interplay between the insulin and lipid metabolism. Insulin is not only critical for control of overall lipid metabolism through its strong ability to suppress lipolysis and ketogenesis and to stimulate lipogenesis, but dysfunctional lipid metabolism, in turn, can suppress the action of insulin.

## Looking to the future

Despite the extraordinary progress in the discovery of insulin and its actions, many of the details about its direct and indirect effects on metabolism remain to be discovered. A new frontier is insulin signaling in the brain. Work by Ron Kahn has shown that insulin action in the brain regulates the cholesterol biosynthetic pathway, and presumably, that mediates many of its systemic actions (129). The interplay between insulin signaling, lipoprotein metabolism, and immune cells has great relevance to inflammatory diseases such as atherosclerosis, one of the major complications of diabetes. The factors that determine the infiltration and differentiation of immune cells in adipose tissue, the vasculature, the liver, and pancreatic islets are rich subjects for future research and are processes in which insulin is likely to play key roles. The events connecting nutrient sensing to insulin secretion in the endocrine pancreas are not fully understood. The full cast of hormones and signaling molecules interacting with pancreatic islets have not all appeared on stage. These are but a few of the exciting areas that will define future discovery in diabetes research.
